# Poisoning with biocidal products before and after the COVID-19 pandemic: report from the Croatian Poison Control Centre

**DOI:** 10.2478/aiht-2025-76-4059

**Published:** 2025-12-30

**Authors:** Emin Veledar, Željka Babić, Jelena Macan

**Affiliations:** Institute for Medical Research and Occupational Health, Division of Occupational and Environmental Health, Poison Control Centre, Zagreb, Croatia

**Keywords:** children, hand sanitisers, insecticides, prevention, rodenticides, surface disinfectants, antiseptici za ruke, dezinficijensi, djeca, insekticidi, prevencija, rodenticidi

## Abstract

Data collected by the Croatian Poison Control Centre (CPCC) in 2020 showed a significant increase in poisoning incidence with surface disinfectants and hand sanitisers compared to the same period in 2019. Considering that this rise in poisoning with biocidal products (BPs) coincided with the start of the COVID pandemic, we wanted to see if there was a trend by analysing six-years’ worth of CPCC records of biocidal poisoning cases covering the COVID-19 pre-pandemic, pandemic, and post-pandemic period (2019–2024). During that period, we received 1320 BP-related calls (8.0 % of a total of 16.441 calls), and 99 % involved four types of BPs: surface disinfectants (542 cases), hand sanitisers (325 cases), insecticides (283 cases), and rodenticides (119 cases). Most poisonings were accidental and involved ingestion and inhalation as the exposure routes. Most patients were asymptomatic or had mild to moderate symptoms, while severe symptoms were mainly observed in suicide attempts, accounting for less than 3.0 % of the cases. Our analysis confirmed an increase in poisoning cases with hand sanitisers during the height of the pandemic (2020–2021) compared to the pre-pandemic 2019, with the numbers somewhat decreasing afterwards (2022–2024), although the number of cases remained higher than before the pandemic. The number of poisonings with surface disinfectants remained similar before and during the pandemic (2019–2021) but showed an increasing trend after the pandemic (2022–2024). Poisoning with rodenticides and insecticides remained stable across all periods. Our findings reinforce the need for greater public awareness of preventive measures, including appropriate labelling and packaging of biocidal products, and for the promotion of safe behaviour in households and workplaces.

## Abstract (hr)

Prema podatcima hrvatskoga Centra za kontrolu otrovanja (CKO), prikupljenima 2020. godine, dezinficijensi i antiseptici za ruke su, kao biocidni proizvodi, prouzročili značajan porast slučajeva otrovanja u usporedbi s istim razdobljem u 2019. godini. U ovom radu provedena je analiza slučajeva otrovanja prouzročenih biocidnim proizvodima, zabilježenih u CKO-u tijekom proteklih šest godina (2019. – 2024.), uključujući razdoblje prije, tijekom i nakon pandemije bolesti COVID-19, kako bi se opisali mogući vremenski trendovi i karakteristike otrovanja. Tijekom promatranog razdoblja zabilježeno je 1320 slučajeva povezanih s biocidnim proizvodima (8,0 % od ukupno 16441 poziva), pri čemu se 99 % slučajeva odnosilo na četiri vrste biocida: 542 slučaja na dezinficijense, 325 na antiseptike za ruke, 283 na insekticide i 119 na rodenticide. Većina otrovanja bila je slučajna, a ingestija i inhalacija bili su najčešći putovi izloženosti. Otrovanja su većinom bila asimptomatska ili s prijavljenim blagim/umjerenim simptomima, a teški simptomi uglavnom su uočeni kod pokušaja samoubojstva, koji čine manje od 3,0 % slučajeva. U razdoblju pandemije (2020. – 2021.) broj slučajeva otrovanja antisepticima za ruke povećao se u usporedbi s 2019. godinom, u postpandemijskom razdoblju (2022. – 2024.) zabilježen je pad, no broj slučajeva i dalje je bio povećan u usporedbi s predpandemijskim razdobljem. Broj slučajeva otrovanja povezanih s dezinficijensima bio je sličan u razdoblju prije i tijekom pandemije (2019. – 2021.), ali je nakon pandemije (2022. – 2024.) uočen trend porasta. Izloženost povezana s rodenticidima i insekticidima nije se mijenjala tijekom promatranog razdoblja. Preventivne mjere, uključujući odgovarajuće označavanje i pakiranje biocidnih proizvoda te promicanje sigurnog ponašanja u kućanstvima i na radnim mjestima, trebalo bi nastaviti.

According to the Croatian Ministry of Health, biocidal products are chemical substances and mixtures containing one or more active substances whose purpose is to destroy, deter, render harmless, or control any harmful organisms or pests in a way that does not involve physical or mechanical action (1). They are divided into four main groups – disinfectants, preservatives, pest control, and other (2) (Table 1) – and can result in accidental poisoning if used and stored improperly in households or workplaces. The Croatian Poison Control Centre (CPCC) discovered concerning household habits, where one-third of parents occasionally stored products outside their original packaging and one-fifth used products intended solely for outdoor use inside the home, often failing to keep them in a safe place out of children’s reach (3). CPCC data collected during the COVID-19 pandemic revealed significant changes in the patterns of disinfectant exposure. In the first half of 2019, surface disinfectants accounted for 1.8 % and hand sanitisers for only 0.4 % of all cases. In the first half of 2020, when the pandemic broke out, these percentages soared: surface disinfectants made up 3.4 % and hand sanitisers 3.8 % of all reported poisoning cases. At the same time, CPCC noted a notable increase in occupational exposure to disinfectants (4) and an overall increase of about 20 % between 2019 and 2024 (5,6,7,8,9,10). A similar trend was reported by the European Association of Poison Control Centres and Clinical Toxicologists (EAPCC) for 21 countries during the first wave of COVID-19 pandemic, showing a significant increase in the overall number of calls and number of calls involving biocidal products for human hygiene (BP1) and disinfectants and algaecides not intended for direct application on humans and animals (BP2) between March and June of 2020 compared to the same interval in 2018 and 2019 (11).

**Table 1:** Types of biocidal products

**Disinfectants**	**Pest control**
BP1 – Human hygiene	BP14 – Rodenticides
BP2 – Disinfectants and algaecides not intended for direct application to humans or animals	BP15 – Avicides
BP3 – Veterinary hygiene	BP16 – Molluscicides, vermicides, and products to control other invertebrates
BP4 – Food and feed area	BP17 – Piscicides
BP5 – Drinking water	BP18 – Insecticides, acaricides, and products to control other arthropods
**Preservatives**	BP19 – Repellents and attractants
BP6 – Storage preservatives for products during storage	BP20 – Control of other vertebrates
BP7 – Film preservatives	**Other biocidal products**
BP8 – Wood preservatives	BP21 – Antifouling products (for vessels, aquaculture equipment, or other structures used in water)
BP9 – Fibre, leather, rubber and polymerised materials preservatives	
BP10 – Construction material preservatives	
BP11 – Preservatives for liquid-cooling and processing systems	BP22 – Embalming and taxidermist fluids
BP12 – Slimicides	
BP13 – Working or cutting fluid preservatives	

Codes 1–22 are taken from the European Chemical Agency (14)

Biocidal products other than disinfectants have rarely been the focus of previous studies, save for those addressing rodenticides and insecticides (12, 13). Therefore, the aim of our study was to analyse all reported cases of poisoning caused by biocidal products over the past six years (2019–2024) to establish and compare their incidence trends and characteristics between the pre-pandemic, pandemic, and post-pandemic period.

## METHODS

We analysed all records of telephone calls to the CPCC, which provides a 24/7 telephone consultation service to the entire population of Croatia (about 3.8 million), and then extracted the data from cases of suspected or symptomatic poisoning in humans by products containing a biocide as an active substance. For products whose trade name was reported, we consulted the Material Safety Data Sheet (MSDS) of the Croatian Institute for Public Health (15) and the Register of Biocidal Preparations of the Croatian Ministry of Health (16) to obtain information about the type and active substances.

The collected data were divided into the four main groups based on product types (Table 1) and analysed in detail for age of the poisoned person, circumstances of exposure, route of exposure, and clinical manifestations. The age groups included infants (up to 23 months old), preschoolers (2–5 years-olds), school children (6–12 years), adolescents (13–17 years), and adults (18 years and older). Symptoms were divided into four categories according to clinical manifestations. The “no symptoms” category refers to cases in which the patient or a parent did not report any symptoms at the time of the call, “mild to moderate symptoms” to those involving mild to moderate digestive tract irritation (vomiting, and diarrhoea) or eye, skin, or respiratory system irritation, “severe symptoms” to serious central nervous system disorders, corrosive damage to the gastrointestinal tract, eyes or skin, severe respiratory symptoms, and “unknown” to cases where symptoms were not specified during the call.

### Statistical analysis

Characteristics of poisoning in regard to patient age, sex, route of exposure, circumstances of poisoning, and clinical manifestations were analysed by descriptive statistics. Significance of differences between the three periods – pre-pandemic (2019), height of the pandemic (2020–2021), and the period following the height of the pandemic (2022–2024) – were tested with the chi-squared or Fisher’s exact test, if the expected subgroup frequencies were <5. Associations were considered statistically significant at P<0.05. All analyses were run on the R Studio statistical software (R Core Team, Boston, MA, USA) (17).

## RESULTS AND DISCUSSION

Between 2019 and 2024, we received 1320 calls reporting human poisoning by biocides. Table 2 breaks them down by year and biocidal product type and shows that disinfectants and pest control products accounted for nearly all biocide poisoning cases (99 %) and that disinfectant poisoning incidence increased steadily by 2024.

**Table 2:** Number of recorded poisoning cases by biocidal product groups and years

**Main groups of biocidal products**	**Pre-pandemic period**	**Height of the COVID-19 pandemic**		**Period following the height of the pandemic**			**Total 2019–2024**	**P value**
	**2019**	**2020**	**2021**	**2022**	**2023**	**2024**		
Disinfectants (BP 1–5) (N)	90	149	132	140	182	174	867	0.002*
Preservatives (BP 6–13) (N)	0	2	3	0	2	3	10	0.278
Pest control (BP 14–20) (N)	69	68	82	71	86	64	440	0.261
Other biocidal products (BP 21–22) (N)	0	0	0	0	2	1	3	0.563
Total number of cases with biocides (N)	159	219	217	211	272	242	1320	0.032*
Total number of reported cases in CPCC (N)	2372	2545	2635	2889	3049	2951	16.441	Not applicable
Percentage of biocides through analysed period)	6.75 %	8.6 %	8.2 %	7.3 %	8.9 %	8.2 %	8.0 %	Not applicable

Codes 1–22 are taken from the European Chemical Agency (14).

* significant differences (P<0.05) between the pre-pandemic (2019), height of the pandemic (2020–2021), and the period following the height of the pandemic (2022–2024) tested with the chi-squared or Fisher’s exact test

Tables 2 and 3 show the fluctuation of calls reporting poisoning with specific groups of biocidal products over the analysed period. Specifically, of 867 disinfectant cases, 325 (37.5 %) involved hygiene products applied to the skin or scalp with the primary purpose of disinfection. In line with our earlier preliminary report (4), the number of reported poisonings with hand disinfectants increased over the pandemic years 2020 and 2021 in comparison to the pre-pandemic 2019, while a mild decrease was seen in the years following the height of the pandemic (2022–2024), although the number of cases remained higher than in the pre-pandemic 2019. The increase from 2019 was significant for both periods that followed Table 3, P<0.001) but we also observed a significant increase between the pre-pandemic period (2019) and the height of the pandemic (2020–2021) (P<0.001), and a significant drop between the height of the pandemic (2020–2021) and the period that followed (2022–2024) (P<0.001).

**Table 3:** Details of poisoning cases by the most prevalent biocidal product groups and years

		**Pre-pandemic period**	**Height of the COVID-19 pandemic**		**Period following the height of the pandemic**			
**Product type**	**Total number of cases**	**2019**	**2020**	**2021**	**2022**	**2023**	**2024**	**P value**
**Disinfectants**	**867**							
Human hygiene	325	13	78	71	47	64	52	<0.001*
Disinfectants and algaecides not intended for direct application to humans or animals	542	77	71	61	93	118	122	0.001*
**Pest control**	**440**							
Rodenticides	119	25	11	22	15	24	22	0.116
Molluscicides, vermicides and products to control other invertebrates	6	2	0	2	0	1	1	0.293
Insecticides, acaricides and products to control other arthropods	283	39	54	53	41	57	39	0.067
Repellents and attractants	32	3	3	4	16	4	2	0.297

* significant differences (P<0.05) between the pre-pandemic (2019), height of the pandemic (2020–2021), and the period following the height of the pandemic (2022–2024) tested with the chi-squared or Fisher’s exact test

Poisoning with disinfectants and algaecides not intended for human use – largely surface disinfectants – accounted for the largest portion of cases in this group of products (62.5 %), and the trend increased significantly in the years following the height of the pandemic (2022–2024) (P<0.001). In other words, while the number of poisonings with hand sanitisers peaked during the height of the pandemic, the increase in poisonings with surface disinfectants occurred with a delay, and is probably the main reason for the observed significant increase in the total number of cases of poisoning with biocidal products (Table 2, P=0.032)

Poisonings with hygiene products were the most common among infants (35.20 %) and preschoolers (29.8 %), followed by adults (27.6 %). Most were accidental poisonings cases (84.6 %), and the most common route was ingestion (85.53 %), while inhalation accounted for only 6.7 % of cases. Asymptomatic cases accounted for 58.7 % of calls, while 39.1 % reported mild symptoms, manifested as mild irritation of the gastrointestinal mucosa. Severe symptoms were reported in a single case of attempted suicide in an adult patient who ingested a large amount of 96 % ethanol, resulting in impaired consciousness.

As regards disinfectants and algaecides not intended for human use, accidental poisonings accounted for 74.4 % and mostly affected adults (55.9 %). Ingestion was the predominant route of exposure (52.39 %), similar to hygiene products, but inhalation exposure was much greater than with hygiene products, accounting for 37.1 %. Most poisonings were reported asymptomatic (52.5 %), while 42.3 % had mild symptoms, most often manifested as mild irritation of the gastrointestinal tract and respiratory mucosa. Severe symptoms were reported in 15 cases. Nine of them involved severe respiratory disorder after accidental inhalation of fumes of chlorine disinfectant and or hydrochloric acid (dyspnoea, wheezing and/or hypoxia, pulmonary oedema, vomiting) or temporary vision loss and headache caused by gluaraldehyde-based fumes. CPCC has repeatedly been receiving such calls describing severe symptoms over chlorine inhalation, because the users mixed products containing hypochlorites and hydrochloric acid to clean the bathroom despite our efforts to raise awareness and educate the general population about the dangers of such practice (18, 19). The six remaining severe cases concerned ingestion, mostly of inorganic acids and alkalis in a suicide attempt, which resulted in gastrointestinal corrosive injury.

A similar trend in disinfectant poisoning was reported by the French Poison Control Centre, which noted a significant increase in exposure to household cleaning products containing biocides and alcohol-based hand sanitisers between 2018 and 2020 (20). The Italian Poison Control Centre reported a significant increase in the number of poisonings with household disinfectants in 2019 and 2020 (21, 22). Kweon et al. (23) analysed the 2017–2021 annual reports issued by the American Association of Poison Control Centers (AAPCC) and had similar findings of hand sanitiser poisonings to ours, inasmuch as they mostly affected children (23). As regards disinfectants not intended for human use, Ghai et al. (24) analysed reports by the California Poison Control System from 2015 to 2021 and, like us, found that adults were affected the most (65 %).

We had no reports of poisoning regarding other types of disinfectants, namely those used in veterinary hygiene, food and animal feed areas, or disinfectants for drinking and potable water (see Table 1).

As regards pest control products, a total of 440 reported poisoning cases were spread more or less evenly across 2019–2024, which suggests no increasing or decreasing trends (Table 3). Most (64.3 %) involved insecticides, acaricides, and products to control other arthropods and most affected children (47.5 %; 25.2 % infants and 22.3 % preschoolers), followed by adults (46.2 %). The majority were accidental poisonings (87.3 %), and 5.7 % were suicidal attempts. Ingestion (63.25 %) was the most common route of exposure, while inhalation accounted for 21.5 %. Most cases of poisoning with insecticides, acaricides, and products to control other arthropods were asymptomatic (61.8 %), 36.4 % caused mild, and four cases had severe symptoms, like intensive vomiting, brief loss of consciousness, and dyspnoea. Three such cases were related to inhalation or ingestion of synthetic pyrethroids (permethrin, alpha-cypermethrin), with developed symptoms. One case involved suicidal organophosphate poisoning with dimethoate-based insecticide.

In the US, Power and Sudakin (13) reported that 35.8 % of all pyrethrin and pyrethroid poisonings affected children between 2000 and 2005, which is in line with our results, just like the share of accidental (93.4 %) and suicidal poisonings (6.54 %). However, their data indicate a yearly increase in exposure to pyrethrins and pyrethroids, whereas our findings show consistent incidence.

In terms of prevalence, second to insecticides/acaricides/other anti-arthropod products were rodenticides with 119 (27.1 %) reported cases, 70.6 % of which were accidental and 14.3 % suicide attempts. They prevailed in children (49.5 %; 21 % in infants and 28.5 % in preschoolers), followed by adults (39.5 %). As expected, the most common route was ingestion (89.1 %), and 78.9 % of cases were asymptomatic, while 20.1 % had only mild symptoms. The only case with severe symptoms was an adult female who ingested an unknown amount of probably concentrated, bromadiolone-based rat poison in liquid form. The patient was found unconscious and admitted to intensive care, where she was successfully treated with vitamin K1 and plasma.

Identical, 49.5 % prevalence of rodenticide poisoning in Norwegian children was reported by Soleng et al. (12) for 2005–2020, and the share of suicidal cases was also similar to ours (15 % in Norwegian report) (12).

We established only six cases of poisoning with molluscicides, vermicides, and products to control other invertebrates, and all were accidental (Table 3).

Accidental were also all 32 poisonings with repellents and attractants for human or animal use, and all were owed to improper storage. Twenty-eight cases involved infants and preschoolers, two involved school children, one an adolescent, and one an adult.

As for other pest control product types, namely avicides, piscicides, and products to control other vertebrates, none were involved in the reported poisonings.

Poisoning with preservatives and other biocidal products was but sporadic across the analysed years (Table 2). Of the 10 reported cases, six involved wood preservatives, and four construction material preservatives. Eight cases were accidental household poisonings, and the remaining two were related to occupational exposure. All involved adults.

As for other biocidal products, all three poisoning cases involved embalming and taxidermist fluids used for the disinfection and preservation of human or animal corpses. All three affected adults through occupational exposure, specifically in a pathological laboratory, where laboratory technicians storing samples inhaled formaldehyde vapours.

The main limitation of our study is that the information on clinical signs and symptoms we received at the time of the call was limited and often provided by a lay caller. This may have diminished the reliability of poisoning severity assessment. During the call, we are focused on as quick and as accurate assessment of health risks as possible. Whether the product is a biocide is not crucial information for patient treatment, so the number of poisonings with biocidal products may have been underreported, as we present only the cases for which we were certain they involved biocides, based on the product name, composition, and description of use provided by the caller.

## CONCLUSION

Regardless of this limitation, this is the first study covering poisoning with all biocidal product types (22 in total). To the best of our knowledge, there are no other reports of a similar scope on post-pandemic trends in poisonings with biocidal products from other countries.

Our results suggest that poisoning with biocidal products remains a public health problem. The most prevalent was the poisoning with disinfectants and pest control product, and we notice an increasing trend in poisoning with surface disinfectants, even after the peak of the pandemic. Hand disinfectants show an opposite, declining trend after the pandemic, but the numbers remain higher than before the pandemic. What raises particular concern is the high share of child poisonings with hand disinfectants, rodenticides, and insecticides, which calls for better labelling and packaging and for restricting the availability of biocidal products in households. We hope that our findings could inform additional preventive measures to reduce the risk of accidents and to ensure safe use of biocides in everyday life and at workplaces.
